# The Influence of Different Acupuncture Manipulations on the Skin Temperature of an Acupoint

**DOI:** 10.1155/2013/905852

**Published:** 2013-02-13

**Authors:** Tao Huang, Xin Huang, Weibo Zhang, Shuyong Jia, Xinnong Cheng, Gerhard Litscher

**Affiliations:** ^1^Institute of Acupuncture and Moxibustion, China Academy of Chinese Medical Science, Beijing 100700, China; ^2^Stronach Research Unit for Complementary and Integrative Laser Medicine, Research Unit of Biomedical Engineering in Anesthesia and Intensive Care Medicine, TCM Research Center Graz, Medical University of Graz, 8036 Graz, Austria

## Abstract

This study was performed to observe the influence of sham and different verum acupuncture manipulations on skin temperature of the stimulated acupoint in healthy volunteers. Thirty-seven healthy volunteers with a mean age of 25.4 ± 2.2 years were enrolled in the study. All volunteers had experienced acupuncture before. They received sham acupuncture and two different kinds of verum acupuncture stimulation (lifting-thrusting and twisting-rotating) on Zusanli (ST36). The skin temperature of ST36 was measured before acupuncture, after needle insertion, after needle manipulation, immediately after removal of the needle, and as further control 5 minutes after removal of the needle using a FLIR i7 infrared thermal camera. During the measurement, the needling sensations of volunteers were enquired and recorded. During the sham acupuncture stimulation, the skin temperature of ST36 decreased in the first 5 minutes, when the point was exposed, and then increased gradually. During verum acupuncture stimulations, the skin temperature increased continually and then decreased in the last phase. The increase in temperature caused by lifting-thrusting stimulation was significantly higher than that of twisting-rotating manipulation, which may be related to the stimulation intensity.

## 1. Introduction

Already in ancient times, doctors realized that acupuncture can influence the skin temperature. This is already mentioned in the first Chinese medical book* Miraculous Pivot,* where it is stated that “the doctor could warm the body through stimulating foot Shaoyin and cool the body through stimulating foot Yangming channels.” So, even at that time it was well known that the skin temperature is an indicator of the reinforcing or reducing acupuncture manipulations. In *Plain Questions*, it is also stated that coldness and heating are part of the indexes of the acupuncture treatment principles.

Around the time of the Jin-Yuan dynasty, the compound manipulations like directional supplementation and draining method (*迎随补泻*, yingsui buxie), heat-producing needling (*烧山火*, shao shan huo), and cool-producing needling (*透天凉*, tou tian liang) were developed to treat diseases aided by heat or coldness feeling after acupuncture manipulation stimulation. These complex manipulations, however, are rarely used in current acupuncture clinic routine; many researchers focus on simpler acupuncture needle manipulations in their experiments and achieve different results.

This experiment tries to observe the influence of Cheng's basic acupuncture manipulations—lifting-thrusting and twisting-rotating—on local acupoint skin temperature in a randomized study, which may lay a foundation for further research.

## 2. Materials and Methods

### 2.1. Selection of Study Participants

Through advertisements on the campuses, 37 healthy volunteers (30 female, 7 male) with a mean age of 25.4 ± 2.2 years (range: 20–35 years) were enrolled among students from the Beijing University of Traditional Chinese Medicine and Graduated School of the China Academy of Chinese Medical Sciences. All participants had received acupuncture before and gave informed consent. The experimental procedure was approved by the Ethics Committee of the Institute of Acupuncture and Moxibustion of China Academy of Chinese Medical Sciences.

### 2.2. Acupuncture

**Figure 1:**
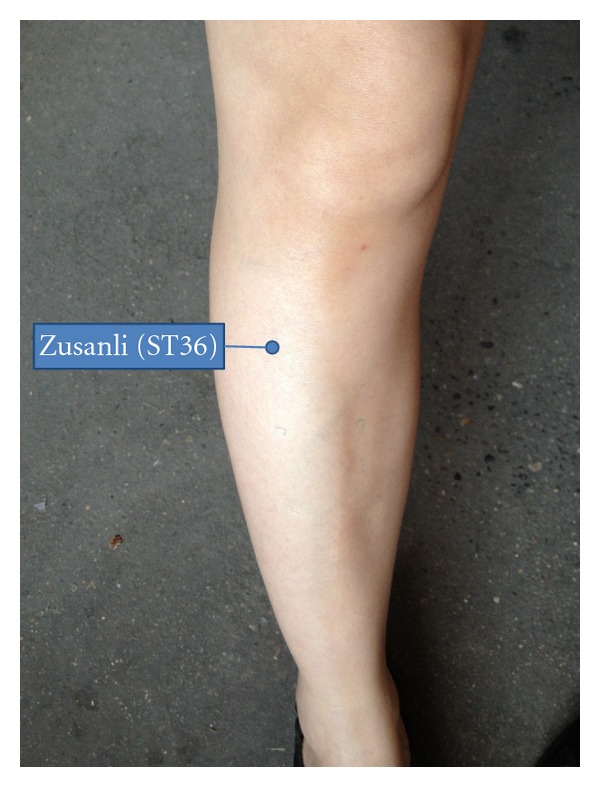
The location of the Zusanli acupoint (ST36).

Each volunteer underwent three measurements (two different kinds of verum acupuncture, see below, and sham acupuncture, in randomized order) every other day. To avoid discrepancies in manipulation, all acupuncture operations were performed by the same medical practitioner. The volunteers lay down on the back and exposed the right lower leg, so the Zusanli (ST36) acupoint could be marked in accordance with a textbook on *Acupuncture and Moxibustion* [1] (see Figure 1). Verum acupuncture was performed according to Cheng [2].

**Figure 2:**
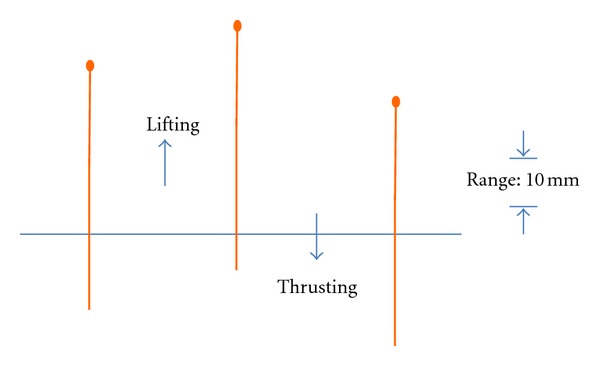
Lifting and thrusting acupuncture needle manipulation.

*Verum Acupuncture: Lifting and Thrusting Manipulation (*提插*, ticha).* Acupuncture stimulation was done manually, using single-use acupuncture needles (0.30 × 40 mm, Zhongyan Taihe brand, Suzhou, China). The doctor inserted the needle on ST36 through a tube to retain depth, until both the practitioner and the volunteer felt the qi arrival; then the insertion was stopped, and the needle remained in place. After 5 mins, the practitioner lifted and then thrust the needle evenly approximately 10 mm (see Figure 2), repeating this operation from 20 to 25 times in 20 seconds. The needle was left in place for 5 more mins and then removed. 

**Figure 3:**
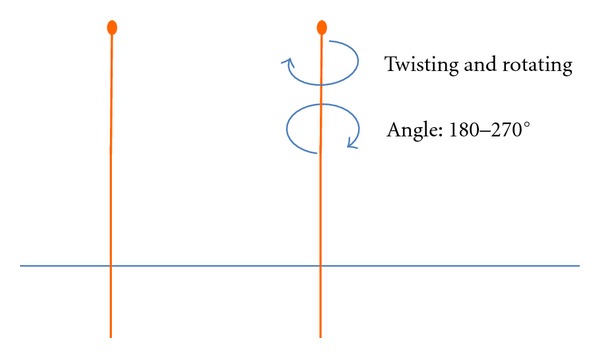
Twisting and rotating acupuncture needle manipulation.

*Verum Acupuncture, Twisting and Rotating Manipulation (*捻转*, nianzhuan).* Acupuncture stimulation was done manually, using the same single-use acupuncture needles as mentioned before. The doctor inserted the needle on ST36 through a tube to retain depth, until both the practitioner and the volunteer felt the qi arrival; then the insertion was stopped, and the needle remained in place (the same procedure as mentioned before). After 5 mins, the practitioner twisted the handle of the needle clockwise and counterclockwise evenly through 180° to 270° (see Figure 3), repeating this operation from 40 to 45 times in 20 seconds. The needle was left in place for 5 more mins and then removed. 

**Figure 4:**
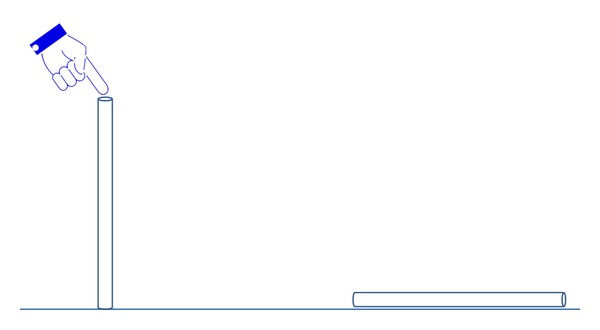
The operation of sham acupuncture.

*Sham Acupuncture (Placebo)*. Sham acupuncture was performed using a single-use acupuncture needle tube (Zhongyan Taihe brand, Suzhou, China) which was tapped on ST36, but no needle was inserted and so the volunteers did not receive any stimulation (see Figure 4).

### 2.3. Measurement of Skin Temperature and Heart Rate

**Figure 5:**
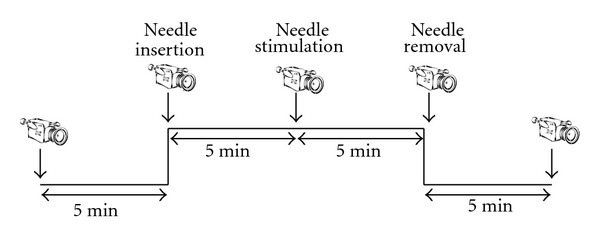
Measurement procedure.

The temperature of the lab was kept at 26°C, and the volunteers were asked to come into the room 5–10 mins ahead of schedule to adapt to the temperature. The skin temperature at ST36 was measured using a FLIR i7 (Flir Systems, Portland, OR, USA) thermographic camera 5 mins before acupuncture, immediately after needle insertion, after manipulation, immediately after removing the needle, and 5 mins after the needle removal (see Figure 5). During the whole experiment, the volunteers' heart rates (HR) were measured with three electrodes on standard positions of the chest, using a Medilog AR12 system (Huntleigh Healthcare, Cardiff, UK).

### 2.4. Needling Sensation

The volunteers were asked about their needling sensation after needle insertion and manipulation, respectively, were assessed by visual analogue scale (VAS). 0 means “no sensation at all,” and 10 means “too much to bear.”

### 2.5. Statistical Analysis

Paired *t*-test was used to compare the temperature changes between the different manipulations, with *P* < 0.05 denoted as significant.

## 3. Results

### 3.1. Changes of HR

Sham as well as verum acupuncture caused changes in the volunteers' HR. After the 5 min phase of rest before acupuncture, HR decreased significantly. After verum acupuncture, needle manipulation, the lifting-thrusting as well as the twisting-rotating stimulation induced a significant increase in HR (compared to the phase of needle insertion), whereas during the sham procedure HR continued to decrease (cf.  Table 1).

### 3.2. Skin Temperature

**Figure 6:**
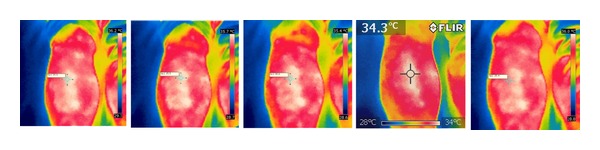
Temperature changes during the sham acupuncture procedure.

**Figure 7:**
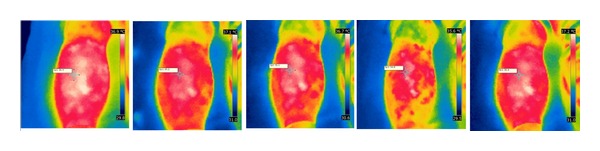
Temperature changes before, during, and after verum acupuncture—lifting-thrusting needle manipulation.

**Figure 8:**
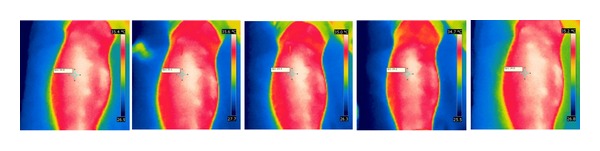
Temperature changes before, during, and after verum acupuncture—twisting-rotating needle manipulation.

After needle insertion, the skin temperature at ST36 decreased insignificantly following the sham procedure, but increased significantly in the two verum procedures. After manipulations, the temperature increase caused by lifting-thrusting stimulation was higher than that caused by sham acupuncture (see Figures 6, 7, and 8).

Table 2 shows the mean and standard deviation of the temperature values of all 37 volunteers.

### 3.3. Needling Sensations during Verum Acupuncture

All subjects could tell sham from verum acupuncture, but could not recognize the lifting-thrusting or twisting-rotating manipulations. After puncturing ST36, 71% of the subjects felt distension, 36% felt sourness, 16% felt pricking, and 10% felt numbness spreading. There was of course no statistical difference in the intensity of the needling sensation when the needle was inserted, but the intensity caused by lifting-thrusting stimulation was significantly higher than that of twisting-rotating manipulation (Table 3).

## 4. Discussion

Previous studies investigating acupuncture and skin temperature used compound reinforcing and reducing techniques to observe changes in skin temperature of the area along the meridian after puncturing the acupoint. A study by Li et al. showed that the skin temperature of acupoints changed according to the frequency of rotating acupuncture stimulation on ST36 [3]. A similar study from Tianjin in China showed that the skin temperature of the abdomen could be increased by reinforcing technique and decreased by reducing acupuncture stimulation technique on ST36 [4]. An experiment by Wang et al. in Shanghai showed that after twisting-rotating acupuncture stimulation with an angle ≤360° on the acupoint SJ5 (Waiguan), the temperature of the ipsilateral acupoint PC1 (Zhongchong) increased [5]. A measurement in Hebei in China showed that after acupuncture stimulation on LI11 (Quchi), the skin temperature of the ipsilateral point LI1 (Shangyang) increased, too [6]. Similar to this experiment, Dong and Che could show that, after electro-acupuncture stimulation on LI4 (Hegu), the temperature of LI4 increased significantly, decreasing slowly afterwards [7].

The lifting-thrusting and twisting-rotating needle manipulations described and investigated in this experiment come from one of the authors' (Professor X. Cheng) clinical experience. His very important results are summarized in *Chinese Acupuncture and Moxibustion* [2]. The clinical technique from Professor X. Cheng has influenced tens of thousands of acupuncturists all over the world. He emphasizes simple and direct clinical acupuncture techniques and uses lifting-thrusting and twisting-rotating manipulations, stopping all operations when qi arrives. These techniques belong to middle and low dosages of stimulations. As his students, the authors adopt his ways in clinical routine and scientific experiments, which allows them to achieve good results [8, 9].

In one of these experiments [9], we used sham acupuncture as a control, considering it to represent the influence of environment, body position, and emotion. It was shown that, even without verum acupuncture stimulation, the skin temperature of the observed point changed significantly. This shows the necessity of a placebo control group in such investigations.

Skin is the only heat dissipation way of the human body; when the body energy metabolism and heat production increases, heat dissipation through the skin increases, too, and so the surface temperature rises [10]. There are three ways of skin heat dissipation: radiation, transmission, and evaporation. Thermal radiation is a procedure of living beings who emit heat in infrared rays to the surrounding environment; the higher the temperature compared to that of the surroundings, the higher the emission of infrared rays. Although we asked the volunteers to come to the lab early to adapt to the temperature, in the first 5 mins the temperature of the observational area decreased, following the exposure. The skin temperature of the acupoint then showed an increase caused by verum acupuncture with deqi arrival, whereas during the sham acupuncture procedure skin temperature continued to decrease. This shows that deqi acupuncture could increase skin temperature, which corresponds to the findings of one of our previous studies [9]. However, 5 minutes are quite short for determining baseline values, and, in further studies, this period should be expanded.

Both twisting-rotating (although not reaching the level of significance) and lifting-thrusting manipulations have been shown to make the skin temperature of the stimulated acupoint increase at first and decrease slowly later on. So, one can say that the lifting-thrusting method causes a stronger needling sensation and a stronger stimulation and induces a higher temperature at the stimulated acupoint. The stronger acupuncture stimulation may be related to the increase in blood perfusion [11, 12].

## 5. Conclusion

Compared to sham acupuncture, deqi acupuncture can be able to increase the skin temperature of the stimulated acupoint. The range of temperature increase caused by lifting-thrusting stimulation is higher than that of the twisting-rotating method. This may be connected with the stimulus intensity. Further research is needed to verify these findings.

## Figures and Tables

**Table 1:** The changes of volunteers' HR (in [1/min]) during the experiment (*P < 0.05).

	Before acupuncture	Needle insertion	Needle manipulation	Needle removal	After acupuncture
Sham acupuncture	71.03 ± 9.04	68.50 ± 8.29*	68.15 ± 7.84	66.79 ± 7.32	67.82 ± 8.04
Lifting-thrusting	73.68 ± 8.88	69.84 ± 9.93*	69.43 ± 8.69	69.95 ± 9.71	71.22 ± 11.01*
Twisting-rotating	69.54 ± 11.47	65.27 ± 12.28*	66.89 ± 10.80	67.32 ± 9.63	67.32 ± 11.50*

**Table 2:** Changes in skin temperature (in [°C]) at ST36. The values in parentheses are the increases (decreases) of temperature with respect to the baseline values (*P < 0.05).

	Before acupuncture	Needle insertion	Needle manipulation	Needle removal	After acupuncture
Sham acupuncture	34.87 ± 1.14	34.69 ± 1.02	35.18 ± 0.67	35.21 ± 1.06	35.23 ± 0.78
	—	(−0.18)	(+0.31)	(+0.34)	(+0.36)
Lifting-thrusting	34.53 ± 1.01	35.01 ± 0.85	35.37 ± 0.79	35.41 ± 1.05	35.35 ± 1.13
	—	(+0.48)	(+0.84)*	(+0.88)*	(+0.82)*
Twisting-rotating	34.49 ± 1.14	34.64 ± 1.38	34.86 ± 1.27	34.96 ± 1.09	34.91 ± 1.14
	—	(+0.15)	(+0.37)	(+0.47)	(+0.42)

**Table 3:** VAS scores expressing the intensity of the needling sensation caused by needle insertion and manipulations (mean ± SD).

	Needle insertion	Needle manipulations
Lifting-thrusting	3.93 ± 1.76	7.13 ± 1.90**
Twisting-rotating	4.72 ± 1.46	4.91 ± 2.32
